# Mitochondrial Factors in the Cell Nucleus

**DOI:** 10.3390/ijms241713656

**Published:** 2023-09-04

**Authors:** Katiuska González-Arzola, Antonio Díaz-Quintana

**Affiliations:** 1 Centro Andaluz de Biología Molecular y Medicina Regenerativa—CABIMER, Consejo Superior de Investigaciones Científicas—Universidad de Sevilla—Universidad Pablo de Olavide, 41092 Seville, Spain; 2 Departamento de Bioquímica Vegetal y Biología Molecular, Universidad de Sevilla, 41012 Seville, Spain; 3 Instituto de Investigaciones Químicas—cicCartuja, Universidad de Sevilla—C.S.I.C, 41092 Seville, Spain

**Keywords:** mito-nuclear crosstalk, stress response, oxidative stress, hypoxia, unfolded stress response, DNA damage

## Abstract

The origin of eukaryotic organisms involved the integration of mitochondria into the ancestor cell, with a massive gene transfer from the original proteobacterium to the host nucleus. Thus, mitochondrial performance relies on a mosaic of nuclear gene products from a variety of genomes. The concerted regulation of their synthesis is necessary for metabolic housekeeping and stress response. This governance involves crosstalk between mitochondrial, cytoplasmic, and nuclear factors. While anterograde and retrograde regulation preserve mitochondrial homeostasis, the mitochondria can modulate a wide set of nuclear genes in response to an extensive variety of conditions, whose response mechanisms often merge. In this review, we summarise how mitochondrial metabolites and proteins—encoded either in the nucleus or in the organelle—target the cell nucleus and exert different actions modulating gene expression and the chromatin state, or even causing DNA fragmentation in response to common stress conditions, such as hypoxia, oxidative stress, unfolded protein stress, and DNA damage.

## 1. Introduction

The mitochondria aare double-membrane organelles where aerobic respiration takes place in eukaryotes. This set of coupled biochemical reactions causes the oxidation of pyruvate to CO_2_, yielding reducing power that fuels oxidative phosphorylation (OXPHOS), in which the chemiosmotic coupling of exergonic electron-transfer reactions drives ATP synthesis. Mitochondria were discovered during the nineteenth century. However, their association with biochemical reactions reported previously—the tricarboxylic acid cycle (TCA), fatty acid oxidation, OXPHOS, and part of the urea cycle, occurred a century later, with the development of cell-fractionation techniques [1,2]. Subsequently, the characterization of most biochemical pathways led to the recognition of mitochondria as the organelles housing the above processes, in addition to other key reactions in essential pathways, such as heme-group [3] and iron-sulphur-cluster syntheses [4], steroidogenesis [5], and the one-carbon cycle [6], which are essential for thymine synthesis from uracil and, therefore, for DNA biosynthesis. The latter routes sustain cellular biosynthetic processes, and the TCA cycle itself supports them by generating reducing power and metabolic intermediates, such as oxalacetate—whose cytoplasmic trans-amination yields aspartate, which is essential to adenosine and pyrimidine nucleotide biosynthesis [7]—and succinyl-CoA, a precursor of δ-aminolaevulinic acid and a key substrate for porphyrin synthesis. The convergence of catabolic and biosynthetic routes makes mitochondria key organelles. This organelle was recognised as a key metabolic hub, in which catabolic pathways meet to fuel cell activity and building blocks are produced to support biosynthetic routes. 

Warburg found hepatocarcinoma cells running aerobic glycolysis, suggesting a mitochondrial connection to pathologies [8] and illustrating the ability of the organelle to take part in coordinated responses [9]. Further, mitochondria were found to modulate intracellular Ca^2+^ concentrations [10]. In the 1970s, calcium was unveiled as a second messenger in many processes, playing key roles in diverse cell processes [11]. These findings provided a rationale involving mitochondrial signalling to explain a wide assortment of pathologies, beyond the alteration of single biochemical reactions. In the 1990s, mitochondria were found to take part in apoptosis. Subsequent well-known milestones are the discovery of the release of cytochrome *c* (Cyt-*c*) to the cytoplasm [12] and the translocation of the apoptosis-induction factor (AIF) to the nucleus [13,14]. More recently, a vast body of works has highlighted mitochondria as a core of cell signals that influence health and aging. A variety of cytosolic sensors of mitochondria-derived signals are known to aim at the nucleus to shift transcriptional patterns [15]. Here, we summarise the role of distinct mitochondrial factors that target the cell nucleus to govern diverse cell responses to stress.

## 2. Mito-Nuclear Crosstalk: An Outcome of Evolution

A large number of genes—*ca.* 1705, including both nuclear and mitochondrial, according to the Human MitoCarta [16]—encode the functionality of mitochondrial responses and their ability to adapt to shifting physiological and pathological conditions. Mitochondria contain multiple copies of a small circular chromosome (mtDNA)—16.569 bp in humans—which limits the loss of this genetic information due to stress, organelle fission, and mitophagy. However, this multi-copy mtDNA comprises 13 protein-coding genes only, together with 22 tRNA genes, two encoding mitochondrial rRNAs and a D-loop region corresponding to a long-noncoding RNA (lncRNA) [17]. Recent reports suggest a large variety of non-coding RNAs (ncRNAs), including small noncoding (snc)-, and circular (c-) RNAs derived from this genome, although their biological relevance remains unclear [18]. Additionally, purported short open reading frames (ORFs) encode at least eight small mitochondria-derived peptides, some of which have emerged as strong metabolic regulators [19]. This tiny chromosomal size results from an intense gene transfer from the parasitic α-proteobacterium to the host and redundancy elimination along the evolution of the endosymbiotic α-proteobacterial ancestry towards a fully integrated organelle [20]. Only some of the nuclear DNA genes encoding mitochondrial proteins derive from the transfer from the endosymbiont, with another non-negligible part derived from other prokaryotes [20,21,22]. Phylogenetic analyses have shown that only 394 of the nuclear genes encoding mitochondrial proteins have a mitochondrial origin, and they were present in proto-mitochondria [22]. This implies that large mitochondrial macromolecular assemblies are indeed mosaics of subunits encoded in distinct genomes. This requires the tight regulation of transcriptional activities in both genomes to synchronise the syntheses of their components [23]. Furthermore, this makes the cell nuclei the main target for signal transduction into transcriptional activity to initiate cellular processes and govern mitochondrial functionality. However, this brings into question how cells respond to each shift in mitochondrial performance. This engages the generation of mitochondrial signals to communicate with cell nuclei, as well as a set of response elements in the latter to trigger cell reaction. Some processes for which human mitochondria are endowed according to gene ontology (GO) searches—like apoptosis, induction or carboxylic-acid metabolism, intracellular transport, or control of cell-redox homeostasis—seemed not to be fully covered by the protomitochondrion proteome [20]. This suggests that host cells furnish mitochondria with gears to fully integrate them along their evolution, in addition to providing essential components to optimise their function as “powerhouses”. 

As proposed by Chandel, a metabolite could be the earliest indicator of mitochondrial operation for the first eukaryotes [15]. Acetate was anticipated as its active derivative—acetyl-CoA—is the substrate for protein acetylation. When targeted to histones, this modification is a key regulator of cell cycle [24,25]. However, new metagenomic analyses—reconstructing the metabolism of the last Asgard common ancestor (LACA)—suggest instead that the host produced acetate, which fed the a-proteobacterium as the substrate [26]. In yeasts, nuclear-cytoplasmic acetyl-CoA synthetase is the main driver of the furnishing of cells with “acetylation power” [27]. In mammal cells, nuclear-cytoplasmic citrate-lyase is the enzyme that transforms excess citrate, produced by the TCA cycle in mitochondria, into acetyl-CoA for later use as a substrate for signalling [25]. Nevertheless, other metabolites may play a part, given the parasitic nature of the pre-mitochondrial ancestor and the use of amino acids as carbon sources by a-proteobacteria, such as *Rickettsia*, to yield dicarboxylic acids [28]. These compounds—α-ketoglutarate (α−KG), succinate, fumarate—can also influence the state of chromatin by regulating the levels of histone modification (see Section 3.5). Further, mitochondrial activity yields reactive oxygen species (ROS) by enzymatic and electron-transport reactions [29]. The ROS levels in cells are tightly regulated by an assortment of mechanisms [29,30,31,32,33], and they play a regulatory role in a broad set of cell processes in mammals, including cell proliferation and mobility [34,35,36]. Therefore, ATP and AMP can be suitable markers of the energetic status of a cell—AMP is already known to regulate AMP-activated protein kinases (AMPKs, see Section 3). Although this is a speculative point, this could be the case for the TACK—*Thaumarchaeota* (*Nitrososphaerota*), *Crenarchaeota* (*Thermoproteota*), and *Korarchaeota*—group of archaea host cells. It is likely that ATP became a signal for mitochondrial-quality control once the relationship evolved and the “guest’s” ATP transporter switched polarity during the transition from parasitic α-proteobacterium to proto-mitochondria. Indeed, it is a key metabolite in the retrograde response (see Section 3) in yeasts [37].

In addition to metabolites, gene-encoded products have also been proposed as putative communicators between the α-proteobacterium and the ancestral archaeal host, as in *quorum* sensing [38].

## 3. From Crosstalk to Integral Signalling

During their evolution, eukaryotic cells have developed housekeeping strategies to maintain mitochondrial functionality and adapt their replication and turnover to physiological needs [39]. The forms of the collection of these regulatory processes can be classified into two groups: one governing the flux of information from the cell nucleus to the mitochondria—called anterograde signalling—and a second, known as retrograde regulation. Nevertheless, both types of control pathway are intimately coupled and knotted to each other. Moreover, they merge themselves with signalling pathways from other cell compartments. In retrograde regulation, upstream signals starting in the mitochondria report their status to the cell nucleus to influence gene-transcription and protein-synthesis profiles. This keeps mitochondrial performance under surveillance, to adapt biochemical routes to the failure of the respiratory electron transport chain (ETC), which is responsible for OXPHOS, and to restore organelle activity. Thus, both the above products of mitochondrial activity and others target primary signal transducers to either elicit or modulate cell responses. 

One example of retrograde regulation is governed by ATP resulting from OXPHOS [40]. In yeasts, ATP targets retrograde response 2 (RTG2) in the cytoplasm, a phosphatase shifting the hyperphosphorylation of the RTG1–RTG3 complex—a transcription factor belonging to the basic helix–loop–helix leucine-zipper family—to its active form by releasing its bound inhibitor, the MKS1–BMH1/2 complex, which is responsible for RTG3 hyperphosphorylation. Upon RTG2 binding, MKS1 dissociates, and it is subsequently degraded [41]. Furthermore, RTG2 also participates in chromatin remodelling, as part of the Saga-like (SLIK) complex, which has histone acetyltransferase (HAT) activity [42]. Notably, a variety of cell-response mechanisms, such as those mediated by the target of rapamycin complex-1 (TORC1) or RAS-cAMP signalling—among others—modulate this apparently simple retrograde control [40]. Thus, ATP default—in mtDNA-depleted (r0^−^, petite) yeasts, for instance—promotes the transcription of genes, allowing the glyoxylate cycle and anaplerotic reactions to complement disrupted TCA cycles, among other responses [40].

A highly conserved cytoplasmic signalling pathway is the AMPK (formerly sucrose non-fermenting 1, SNF1) route, involving ADP and AMP as elicitors [43]. This pathway is active when OXPHOS is unable to support efficient ATP synthesis, which leads to increases in both AMP and ADP. In mammals, the activation of AMPK requires its phosphorylation by either the Ser/Thr liver kinase B1 (LKB1, a.k.a. STK11) [44]—in neurons—or the calcium/calmodulin-dependent protein kinase 2 (CAMKK2) [45,46]. Thus, AMPK is additionally upregulated by other factors, such as cytoplasmic calcium levels, overall energetic homeostasis, and different sources of stress by cytoplasmic signalling pathways affecting post-translational modifications (PTMs) cascades, chromatin remodelling, and gene-expression patterns.

In mammals, the G-protein pathway suppressor 2 (GPS2) has been suggested as a functional analogue of RTG2 [47]. The GPS2 is a mainly nuclear-cytoplasmic protein, acting as the master regulator of a variety of processes, such as inflammation [48], insulin signalling—via the AKT (protein kinase B) route [49]—and cholesterol regulation [50], among others. Knockout GPS2 is lethal to mice embryos. The GPS2 controls the expression of nuclear-encoded mitochondrial genes, in addition to others involved in Golgi, endoplasmic reticulum (ER) and lysosomal function, cytoskeletal performance, or chromosomal management [47]. Notably, there are two minor populations of GPS2 in the mitochondria: one in the matrix and another in the outer mitochondrial membrane (OMM). These two species also differ in their PTMs, with the OMM species purportedly displaying SUMOylation. Upon ETC decoupling, these mitochondrial species migrate to the cell nucleus, causing a new transcriptional response that is different from others, which are mediated by the protein. The mechanism leading to this differential gene-expression pattern is puzzling, since it is unclear that PTMs are key for the translocation of these minor GPS2 species. Therefore, the specific roles played by these mitochondrial species within the organelles need further clarification.

Research on a wide variety of pathologies has revealed a highly complex landscape of retrograde regulation routes and their effects [39,51,52,53,54,55,56]. In addition, many toxicants target the mitochondria—either in a direct manner or indirectly—causing their failure, again affecting organs with strong energy needs [57]. The causes of these conditions can be of external origin—such as bacterial toxins—or internal, such as hypoxia, stroke and reperfusion, inflammatory processes, and cell-state transitions such as the endothelial–mesenchymal transition (EMT) and tumour development, among others. Often, severe mitochondrial alterations exert pleiotropic effects within and outside the organelle, leading to common dysfunctional features, such as mtDNA damage and copy-number decrease, the loss of protonmotive force (ΔΨ) and Ca^2+^ retention capability, OXPHOS and TCA impairment, protein-import loss, increased ROS production, altered levels of metabolites, mitochondrial segmentation, etc. Upon their detection, a cascade of signal transduction takes place, causing protein PTMs, chromatin remodelling, and changes in gene-expression patterns. The redundancy of signal mediators and feedback regulation causes these response networks to show dual stability, which converts a continuous range of stimuli in a binary response [58]. Thus, mitochondrial dysfunction generates responses that lead to either the recovery of physiological cell operation or pathology, according to the concept of mitohormesis. For instance, an acute stimulus causes the unfolded protein response (UPR) to trigger apoptosis, while a mild stimulus leads to adaptation [59]. 

Although different conditions cause initially distinct mitochondrial responses, the signalling pathways may blend into a more general response. For instance, the reaction to hypoxia affects inflammatory responses through ROS signalling, as well as affecting the ER and mitochondrial UPRs (UPR^ER^ and UPR^mt^, respectively) [60,61]. Moreover, a single mitochondrial factor can partake in or modulate responses to distinct stimuli by alternative routes. Nevertheless, in each, we can distinguish one or more mitochondrial factors acting as messengers to other organelles and cell nuclei.

### 3.1. Adaptation to Oxygen Levels and Mitochondrial Regulation of Nuclear-Gene Transcription

Although it poses the risk of oxidative damage, oxygen is an essential substrate for OXPHOS. Hypoxia is a common stress in pluricellular organisms, resulting from the imbalance between the supply and the demand of oxygen [62]. In cell cultures during normoxia—with proper airing—oxygen levels are *ca.* 21% (160 mmHg). However, oxygen concentrations drop to an average steady-state level of 6.1% under physiological conditions, in which arterial blood irrigates peripheric tissues (physoxia) [62,63]. Physiological hypoxia are situations in which cell responses can stimulate a return to homeostasis. This is related to the physoxic levels in each tissue, laying on the range of 2% oxygen. Finally, the persistence of poor oxygenation leads to pathological hypoxia, often with O_2_ levels below 1%.

#### 3.1.1. Response Mediated by Cytoplasmic and Nuclear Factors: HIF1α

**Figure 1:**
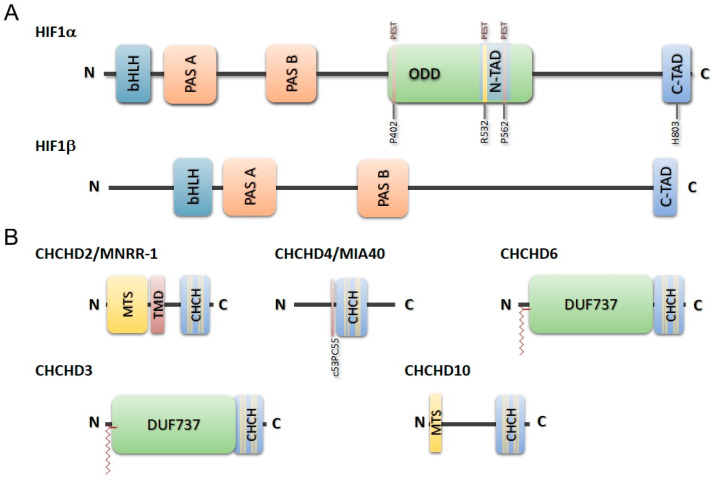
Proteins involved in the response to oxygen levels. (**A**) Schema of HIF1α and HIF1β. bHLH stands for basic helix–loop (turn)–helix domain, PAS stands for Per-Arnt-Sim domains, and ODD is the oxygen-dependent degradation region. Key sequence stretches (PEST) and residues (P402, R532, and P562) for HIF1α regulation in the ODD. Representation also shows the location of the two transactivation domains (N- and C-TAD). (**B**) Representative members of the coiled-coil helix coiled-coil helix domain in this work (see page 7). CHCH domains are in blue, with yellow bars showing the approximate locations of CX_9_C motifs. MTS stands for mitochondrial targeting sequence, TMD is a transmembrane domain, DUF stands for domain of unknown function. Red acyl groups indicate glycine myristylation sites.

Both anterograde and retrograde routes govern hypoxia responses. One signal route involves the oxygen-dependent activity of the hypoxia-inducible factors 1 (HIF-1) and 2 (HIF-2). These are basic heteromeric transcription factors containing a basic DNA-binding helix–turn–helix (bHLH) Per-Arnt-Sim (PAS) monomer (HIF1α, Figure 1A) [64]—similar to its paralogs, HIF2α [65] and HIF3α [66]—and a second unit, the aryl hydrocarbon receptor nuclear translocator (ARNT) domain (HIF1β), which also contains a basic helix-turn-helix, PAS [64]. The HIF-1α is ubiquitous, whereas the other isoforms are rather cell-specific, and differ in their specific interactions [67]. In addition, HIF-1 is constitutively expressed, and it is normally located in the nucleoplasm, so it cannot be considered a mitochondrial factor. Nevertheless, some reports indicate that a minor fraction (1–5%) of the protein may be located in mitochondria under oxidative stress or hypoxia [68,69]. Notably, the α-subunit includes an oxygen-dependent degradation region (ODD) containing a N-terminal trans-activation domain (N-TAD) comprising two PEST—proline, glutamic, serine, and threonine—sequences, which are responsible for proteasomal degradation in the presence of oxygen [70,71]. 

The degradation of HIF1α requires the post-translational hydroxylation of proline residues by prolyl-4-hydroxylases (PHDs)—Fe^2+^-dependent dioxygenases using oxygen and α-KG as substrates, and ascorbate as an electron donor [72]. This regulatory mechanism is conserved among metazoans. The hydroxylation of PEST enables interactions with the von Hippel Lindau (pVHL) E3 ligase component, ubiquitination, and proteasomal degradation. The Michaelis–Menten constant (*K*_M_) values of the PHDs for O_2_ are *ca*. 230 mM—*ca* 6.3% O_2_ in solution, matching the levels in physoxia [62,63,73]. Active HIF1α binds to the β-subunit, and the active heterodimer promotes transcription from core DNA sequence (G/ACGTG) in hypoxia response elements (HREs) coupled to several target genes [74,75]. Notably, PHDs are inhibited by TCA metabolites, such as fumarate (*K*i 50–80 μM), succinate—apyruvate dehydrogenase (PDH)-reaction product (inhibitory constant, *K*i, *ca*. 400 μM)—and citrate, with a lower affinity [76]. Thus, mutations in TCA enzymes, such as succinate dehydrogenase (SDH) or fumarate hydratase (FH), can lead to anomalous, pseudo-hypoxic cell responses, as observed in different types of cancer.

The HIF1α activity is further regulated, in a pVHL-independent manner, by hydroxylation in its carboxyl terminal-trans-activation domain (C-TAD) by asparaginyl hydroxylase (FIH), which prevents binding to p300, the cAMP-response element-binding protein (CREB)-binding protein (CBP), or other transcriptional coactivators [77,78]. These proteins bind the C-TAD of HIF1α through their first cysteine–histidine-rich domain (CH1, a.k.a. TAZ1) [79,80], which was initially considered essential for the transcription of HIF target genes. Furthermore, CH1 participates in roughly 48% of HIF-mediated transcriptional activities, including those involving the participation of histone deacetylase 7 (HDAC7) [79,81]. This is surprising, since CBP and p300 have a bromodomain to bind acetylated histones and the CH2 domain shows histone acetyltransferase (HAT) activity. Authors suggested that acetylation and deacetylation could take place at separate times. According to the Reactome site [82], more than a hundred genes display HRE elements. 

Oxygen levels further regulate HIF1 activity through the expression of the HIF3α locus. First, the promoter of this gene displays an HRE sequence targeted by HIF1 itself [83]. Next, transcripts from this gene are subjected to an alternative splicing mechanism, which is oxygen-dependent [84]. The alternative transcript encodes a PAS protein (IPAS) that binds to and inhibits HIF1α. Further, oxygen also regulates the expression of the CBP/p300-interacting trans-activator 2 (CITED2) via cytoplasmic Fork-head box O-3 (FOXO-3) activation [85]. In its turn, CITED2 is an allosteric competitor for CH1 binding to HIF1α [86]. Additionally, HIF1α controls the expression of a plethora of ncRNAs with effects on a broad set of processes, many of which control HIF1α expression and activity at different levels [87,88]. 

The dependence of HIF activation on the activity of PHDs mixes the anterograde character of this route with retrograde signals. This may result from bottleneck in HIF regulation created by PHD kinetics, as highlighted in the literature [89]. First, mitochondrial dysfunction—e.g., OXPHOS impairment—leads to the inhibition of complex II (CII or succinate dehydrogenase, SDH) and, therefore, of the TCA cycle. This leads to succinate depletion and α-KG accumulation. The first is one of the products of PHD-catalysed reactions and an inhibitor of the enzyme, while the second is its substrate, thereby exerting contrasting effects on PHD activity, on HIF1α stability, and on HIF assembly. Indeed, mutations in FH and SDH promote HIF-hydroxylation activity [90]. This results in the expression of genes shifting energy metabolism and favouring glycolysis, affecting cell proliferation and survival and iron homeostasis, among other processes, depending on the cellular context. Similarly, the iron cofactor of PHDs is sensitive to ROS, and it eventually requires ascorbate to exit non-functional oxidised states [91]. In addition, since HIF is sensitive to oxygen, ROS, and the redox state, its interactions with a wide variety of coactivators are also affected [62]. Thus, oxidative stress mediates another retrograde regulation of HIF-mediated response to mitochondrial dysfunction. 

To our knowledge, cytochrome *c* oxidase (complex IV, CIV, a.k.a. COX) is the only enzyme in cells that uses oxygen directly for energy transduction. During hypoxia, its activity transiently increases, before declining as glycolysis takes over cell metabolism. A transcriptional screening revealed three genes showing early and transient upregulation upon hypoxia [92]. One of these genes encodes the hypoxia-inducible gene-domain-family member 1A (HIGD1A)—a homologue of yeast respiratory super-complex factor 1 (RCF1)—whose product binds CIV to promote super-complex formation [92,93]. The HIGD1A is transiently induced under hypoxic conditions and increases the activity of complex IV, leading to increased oxygen consumption and subsequent mitochondrial ATP synthesis, thereby improving cell viability during hypoxia [92]. Recent data show that HIGD1A and its homolog, HIGD2A, participate in the assembly of CIII and CIV [94]. Further, its expression was previously detected in a hypoxic brain by microarray screening [95]. In addition, HIGD1A is already known to protect cells from low glucose and hypoxia [96]. The HIGD1A gene is activated by HIF in a CH1-independent manner [97]. In glioma cells, residual HIGD1A levels are maintained separately from HIF induction, and linked to the activity of the DNA methyl transferase-1 (DNMT1) on HIGD1A HREs [98]. Although HIGD1A is more present in the brain, the expression of several of its homologs takes place in other organs. All its homologs are targeted to the intermembrane spaces of the mitochondria and exert a protective effect, impairing the release of Cyt-*c* into the cytoplasm, among other effects [99]. In cardiomyocytes, this protective effect also involves an increase in CIV activity [92]. Notably, Cyt-*c* phosphorylation modulates improvements in CIV performance by HIGD1A [100,101]. Nevertheless, co-immunoprecipitation experiments suggest that HIGD1A is rather in contact with complex III (CIII) [98]. 

#### 3.1.2. Mitochondrial Factors and Responses to Oxygen Levels

**Figure 2:**
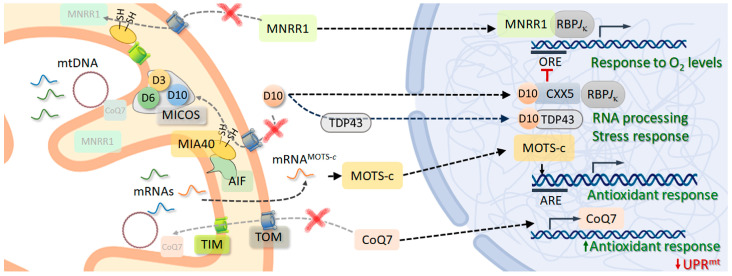
Transcriptional regulation of nuclear genes by mitochondrial proteins in response to perturbations in oxygen metabolism. All these proteins, except MOTS-c, are nuclear-encoded. Inhibition of the import system by low oxygen or oxidative stress leads to the accumulation of otherwise mitochondrial factors in the cell nucleus, activating different responses according to target promoter and modulator. ORE stands for oxygen-responsive element; ARE stands for antioxidant response element. Proteins of the CHCHD family, except MIA40, are abbreviated as D3, D6, and D10. MOTS-c transcription is fostered by oxidative stress, and the transcript is translated in the cytoplasm. TIM and TOM stand for the inner and outer membrane-transport systems, respectively. MICOS stands for mitochondrial contact site and cristae-organizing system, which, together with the F_1_ F_O_-ATP synthase, and optic atrophy 1 (OPA1) protein, facilitates formation, maintenance, and stability of cristae membranes.

The hypoxia response also involves alternative—albeit complementary—pathways, acting under less severe conditions. A mitochondrial gene whose product is usually located in the intermembrane space (IMS)—the mitochondria nuclear retrograde regulator 1 (MNRR1, a.k.a., coiled-coil-helix-coiled-coil-helix domain 2, or CHCHD2)—was detected through a computational gene-expression search for MIA40 interactors across the NCBI Gene Expression Omnibus database [102]. This protein, together with the mitochondrial intermembrane-space import and assembly protein 40 (MIA40, a.k.a., CHCHD4, a key factor in mitochondrial protein import), CHCHD10, and others, presents helix–turn–helix domains with twin CX_9_C motifs, enabling the formation of interhelical disulphide bonds characteristic of the CHCHD-protein family (Figure 1B) [103]. The MNRR1 uses the MIA40 import system to enter mitochondria, despite presenting a mitochondrial targeting sequence in its N-terminus [104]. Mild hypoxia impairs this import pathway, so MNRR1 accumulates in the cell nucleus (Figure 2) [105]. Notably, MIA40 mediates imports linked to cysteine oxidation, suggesting that the conformation of nuclear MNRR1 differs from that of its nuclear species. In fact, the substitution of cysteine residues from the CX_9_C motifs by serine yield the retention of this protein in the cell nucleus [104]. However, there are some intriguing aspects in the localization of these proteins, such as the folding state of the nuclear population. If it is folded, it is questionable whether this folding is due to the activity of extramitochondrial chaperones.

Knocking MNRR1 down decreases CIV-turnover rates by *ca*. 40% in cells [104]. The MNRR1 co-purifies with COX, a interaction that is enabled by ABL-related gene (ABL2) phosphorylation within the IMS [106]. The MNRR1 levels increase in the mitochondria in response to the dissipation of ΔΨ—not ΔpH—across the IMM through a hypoxia-insensitive mechanism involving PTMs [107]. Recently, MNRR1 was shown to modulate mitochondrial fusion by controlling the processing of the optic atrophy 1 product (OPA1) by YME1 Like 1 ATPase (YME1L1) protease. First, the YME1L1-encoding gene is under the control of an oxygen-responsive element (ORE) targeted by MNRR1 [104,108]. In addition, MNRR1 binds to complement component 1 Q subcomponent-binding protein (C1QBP), which modulates YME1L1 protease activity [109]. 

Hypoxia elicits MNRR1 expression, which, in turn, activates tissue-specific COX subunit 4 isoform 2 (COX4I2) transcription in the cell nucleus. This involves binding to a 13-base-pair ORE in its promoter [108,110]. These elements comprise four out of five nucleotides in the HRE sequence—the fifth being T in place of G—but seem to be unresponsive to HIF1α regulation [108]. The MNRR1 competes with the CXX5 repressor to bind recombination-signal-binding protein for the immunoglobulin kappa J region (RBPJκ) transcription factor, which then activates transcription from ORE [108]. Moreover, the MNRR1 gene has an ORE in its own promoter; thus, it is activated by its own product. 

In addition, MNRR1 is also under transcriptional regulation by the cAMP-responsive element-binding protein (CREB 3-like protein 3), a fact that links MNRR1 expression to both the UPR^ER^_,_ and the UPR^mt^ [105]. These processes contribute to quality control (QC) tasks that are key for cell survival. Furthermore, MNRR1 lifetimes depend on the proteolytic activity of YME1L1 and OMA1 zinc metallopeptidase [111]. Analyses with KO models indicate that UPR^ER^ causes UPR^mt^ in an MNRR1-dependent manner, upstream of known UPR^mt^ effectors such as the basic Leu-zipper activating transcription factor 5 (ATF5)—the mammal homologue of *C. elegans* ATFS-1. In mitochondrial myopathy encephalopathy, lactic acidosis, and stroke-like episodes (MELAS) cybrids, MNRR1 levels are low, and UPR^mt^ is defective [105].

The dysfunction of another protein in this family, CHCHD10 (Figure 1B), emerged recently as a relevant factor in neurodegenerative diseases, such as amyotrophic lateral sclerosis (ALS) and ALS-frontotemporal dementia (ALS-FTLD) [112,113,114], among other neurodegenerative diseases. The CHCHD10 and MNRR1 proteins display 58% sequence identity, and they probably evolved from a gene-duplication event [115]. More recently, it was proposed that this protein modulates mitochondrial functionality in several tissues by affecting mitochondrial morphology, the steadiness of mitochondrial cristae, CIV activity, mtDNA stability, and cell survival under stress. Furthermore, CHCHD10 is a 14 kDa protein that is also located in the IMS—weakly bound to the IMM [116]. Together with mitofilin, CHCHD3, and CHCHD6, it has been proposed, CHCHD10 takes part in the mitochondrial contact site and cristae organizing system (MICOS) system, which determines the IMM topology and the formation of membrane-contact sites [117]. However, the relevance of CHCHD10 to MICOS stability is a controversial issue. Mutagenesis directed by CHCHD10 affects mitochondrial IMM shapes in fibroblasts [114], also observed in specific FTD-ALS patients [113], but not in KO mice tissues [113]. Fibroblast mitochondria from ALS patients displaying CHCHD10 mutations have altered nucleoids, with limit repair ability, since the recovery after H_2_O_2_ treatment is only 60% compared to when wild-type controls are used. The permeabilization of the OMM (MOMP) is also affected in these patients. Furthermore, CHCHD10 expression is higher in tissues with high energy demand, such as the skeletal muscles, heart, liver, kidneys, and substantia nigra [116]. With the exception of the heart, CHCHD10 KO hardly affects OXPHOS performance, but instead increases the iron levels within the mitochondria. As with other twin CX_9_C-motif-containing proteins, the entry of CHCHD10 into the mitochondria is MIA40 (CHCHD4)-dependent, and Cys-to-Ser mutations impair its import into the IMS. In addition, MNRR1 expression takes place at O_2_ levels as low as 4% [108], while CHCHD10 transcription occurs in a narrow interval at higher concentrations (8%), proposed to be the physioxic level.

As with MNRR1, CHCHD10 can be detected mainly in mitochondrial cristae and, then, in the nucleus [118]. The functions of both proteins are tightly linked, with CHCHD10 acting as a scaffold to enable MNRR1 phosphorylation within mitochondria, and forming an MNRR1-mediated complex with CIV, stimulating its activity. In the cell nucleus, contrary to MNRR1, CHCHD10 acts as a repressor in the ORE sequences by interacting with the CXX5 repressor, and morbid mutations of CHCHD10 impair these interactions [119]. Nevertheless, CHCHD10 KO does not affect MNRR1-transcription levels [118]. Thus, at physioxic concentrations [50], transcription from ORE-containing promoters is inhibited by CHCHD10.

Again, CHCHD10 protein levels are proteolytically controlled by YME1L1 and OMA1 proteases, suggesting their participation in UPR^mt^ (see Section 3.3). Uncouplers such as carbonyl cyanide m-chlorophenylhydrazone (CCCP) cause the accumulation of both MNRR1 and CHCHD10, mainly in the mitochondria, but also in the cell nucleus and, probably, in cytosol [107,111]. As with MNRR1, in the mitochondria, such increases are due to decreases in their proteolysis. Notably, the MIA40/ERV1 (CHCHD4/ALR in humans) import system is non-responsive to ΔΨ changes, whereas the mitochondrial proteases, OMA1 and YME1L1, are degraded upon IMM depolarization [120]. 

In addition, CHCHD10 binds TAR DNA-binding protein 43 (TDP43) in the cell nucleus upon IMM depolarization [118]. This protein belongs to the family of heterologous ribonucleoproteins (hnRNPS) and is key to RNA metabolism. In addition, TDP43 acts both in the cell nucleus—regulating transcription, splicing, miRNA biogenesis, and the binding of lncRNAs—and within cytosol, influencing mRNA stability, trafficking, and translation [121]. The binding of CHCHD10 modifies the cellular location of TDP43, targeting it to the cell nucleus. Several ALS-FTD-associated CHCHD10 mutations (R15L, S59L) result in TDP43 accumulation in cytosol, a substantial amount (>35%) of which is located in mitochondria and speckles, with a decrease in TDP43 content in stress granules [118,122]. This resembles the effect of a reported ALS-related gain-of-function mutation (S55L), which causes the aggregation of CHCHD10 itself within mitochondria and triggers the integration of UPR^mt^ [114].

To summarise, there is a tight link between O_2_ levels, ETC operation, IMM proton-motive force, and other features of the cellular functional state, such as the UPR. The responses to their changes are partly mediated by the performance of several mitochondrial proteins as activatable transcription factors in the cell nucleus.

### 3.2. Dealing with Oxidative Stress

Oxygen reactive species (ROS) and the products of their reactions with other cellular or mitochondrial components also act as signal triggers. The fortuitous electron leakage associated with the activity of the ETC—and TCA cycle enzymes, among others—makes the mitochondria the main generators of these species [29,123]. The activity of diverse enzymes in mitochondria, cell membranes, peroxisomes, the endoplasmic reticulum, and even the cell nucleus is an additional source of ROS [124]. This includes radicals such as superoxide anions (O_2_^−•^), hydrogen peroxide (H_2_O_2_), and non-radical, oxidising compounds, such as singlet oxygen (^1^O_2_), ozone (O_3_), and organic peroxides (ROOH). In addition, superoxide anions may react with nitric oxide (NO^•^) to yield reactive nitrogen species (RNS), such as peroxynitrite (ONOO^−^). These reactive species can alter different kinds of biomolecule according to their chemical nature [125,126,127]. The ROS and RNS can indeed cause up to 20 amino-acid modifications in proteins, in addition to affecting cofactors such as iron–sulphur clusters [128]. Such PTMs either play a physiological, regulatory role, or constitute a pathological burden, depending on the target. Control over ROS levels requires the action of small antioxidant molecules and enzymes [129]. The latter include superoxide dismutase (SOD) isoforms, glutathione peroxidase (GPXs), catalase (CAT), and glutathione reductases (GR), among others. In addition, the activity of uncoupling protein 2 (UCP2) modulates ROS production by the ETC under stress in mammal brains, as reviewed by Shadel and Horvath [130]. Such modulation is also key to control over adaptive, concerted tissue responses, in which ROS act, for instance, as signalling transmitters for “the synchronization of neural circuit activity” [130]. Cell housekeeping demands the adaptation of all these activities to actual needs, again implying the need for sensors and associated signal pathways. 

The dysregulation of ROS levels causes oxidative stress, leading to excess lipid peroxidation, protein modifications, and DNA damage. Excessive oxidative stress leads to a variety of cell-death processes, including apoptosis and ferroptosis. Cell death is beyond the scope of this text, so we address it only partially. Nevertheless, it is worth mentioning that apoptosis involves the release of mitochondrial factors into the cell nucleus and the cytoplasm. Two IMS proteins target the cell nucleus and partake part in the assembly of the DNA fragmentation factor (DFF) complex: a c-terminal truncated form of AIF (_t_AIF) and Endonuclease G (ENDOG) [131,132]. Typically, their release during apoptosis is caspase-dependent, since the mitochondrial outer-membrane permeabilization (a.k.a. MOMPT) event is insufficient [133]. The DFF complex, also comprising caspase-3-activated DNase (CAD) from a cytoplasmic origin, is responsible for DNA degradation during apoptosis [134]. Notably, both proteins can also mediate caspase-independent DNA fragmentation, even when _t_AIF lacks nuclease activity. It has been proposed that nuclear _t_AIF (i) increases the susceptibility of DNA to latent nucleases, (ii) recruits downstream nucleases, and (iii) disrupts chromatin structures by displacing chromatin-associated proteins [135,136]. Furthermore, _t_AIF nuclear redistribution is observed in different species during some types of cell death [137]. For instance, nuclear _t_AIF is detectable in neuronal apoptosis induced by retinal detachment [138], brain trauma [139], cerebral ischemia [140], exposure to *Streptococcus pneumoniae* [141], hydrogen peroxide, peroxynitrite [139], or infection with a p53-expressing adenovirus [142].

AIF is a flavin adenine dinucleotide (FAD)-containing protein that was initially reported as being located in the mitochondrial intermembrane space [135]. Instead, AIF is attached to the inner membrane, with its C-terminus at the IMS [143]. It binds two FAD molecules and one nicotinamide adenine dinucleotide (phosphate) or NAD(P), responsible for AIF-redox activities. Under physiological conditions, AIF-redox activity is essential for optimal oxidative phosphorylation, enabling the assembly of respiratory complexes I and III [144]. In addition, AIF dysfunction is related to increased ROS levels [145,146]. In fact, AIF binds CHCHD4 (Figure 2), thereby taking part in the redox-dependent mitochondrial import-and-folding system for nuclear-encoded small proteins containing cysteine-rich motifs [147]. This could explain why AIF deficiency leads to severe mitochondrial dysfunction, causing muscle atrophy and neurodegeneration, associated with at least 19 different syndromes [148,149]. Originally, AIF was discovered as an apoptosis-inducing protein, with a caspase-independent role as a cell-death effector [13]. Pro-apoptotic stimuli cause the proteolytic cleavage of AIF, so its C-terminal domains are released [143]. Processed _t_AIF must enter the nucleus to promote cell death [150]. Furthermore, _t_AIF causes chromatin condensation and the large-scale fragmentation of DNA to fragments of about 50 kbp [135], typical of apoptosis. Mutations that abolish _t_AIF–DNA electrostatic interactions suppress _t_AIF-induced chromatin-condensation activity [150,151]. In addition to forming DFF, _t_AIF interacts with as histone H2AX [152] and cyclophilin A (CYPA) [153]. Interactions between _t_AIF and CYPA take place in the cytoplasm, and they are co-transported to the cell nucleus. Neither of the two proteins has nuclease activity, but their complex can degrade DNA, leading to caspase-independent DNA fragmentation. Additionally, AIF causes the redistribution of other apoptogenic factors, such as Cyt-*c* and pro-caspase 9. Further, the microinjection of AIF into the cytoplasm of intact cells induces well-known apoptotic hallmarks: mitochondrial ΔΨ dissipation and the exposure of phosphatidylserine in the plasma membrane [135].

Oxidative stress also enhances the expression of the nuclear ENDOG gene [154]. The ENDOG gene belongs to the family of DNA/RNA non-specific ββα-metal-finger nucleases [155]. It is also located within the IMS. The ENDOG participates in the replication, recombination, and removal of damaged mitochondrial DNA [156,157]. For this purpose, it relocates from the IMS to the matrix, where it digests mitochondrial DNA, promoting paternal mitochondrial elimination [158]. Nevertheless, during apoptosis, ENDOG is released from the mitochondria and translocates to the cell nucleus to induce GC-rich DNA fragmentation, independently of caspases [131]. In the nucleus, this nuclease can also digest double-stranded or single-stranded DNA and DNA–RNA heteroduplexes [159]. In addition, ENDOG can drive caspase-independent cell death under stress [160]. 

The release of ENDOG from mitochondria is mediated by the truncated form (tBID) of BH3 interacting domain death agonist (BID)—a pro-apoptotic member of the B-cell lymphoma 2 protein family(BCL-2, described below) that is critical for death-receptor-mediated apoptosis [161]. The BCL-xL—an antiapoptotic member of this family known to interact with the import system—prevents this activity. Moreover, the C-term of the HSC70-interacting protein (CHIP) regulates the apoptotic activity of the endonuclease in a HSC70-dependent manner [154]. Both facts suggest the relevance of the mitochondrial import system in ENDOG-mediated cell death. Further, they constitute another link between protein-quality control, UPRs, and mitochondrial responses to stress. In contrast, ENDOG has been reported to perform a protective role in the cell nuclei of ovarian cancer (OC) cells, which, it has been suggested, involve its interaction with Aurora B [162]. In OC cells, the nuclear location of ENDOG requires the activity of cIAP1 E3-ligase and elevated ROS levels. We speculate that high ROS may cause protein oxidation, affecting ENDOG activity. Thus, ENDOG is crucial during early embryogenesis, but its nuclease activity is dispensable for both embryogenesis and apoptosis [159].

In addition to its role in the cell nucleus, ENDOG is a crucial regulator of autophagy in multiple species, and it is also involved in the DNA-damage response [163]. In response to starvation, glycogen synthase kinase 3 beta (GSK-3b) phosphorylates ENDOG at Thr-128 and Ser-288, enhancing ENDOG’s interaction with 14-3-3γ [163]—a member of the 14-3-3 family of phospho-binding proteins that regulate cellular functions such as apoptosis, cell-cycle progression, and autophagy [164]. Thus, ENDOG competitively binds to 14-3-3γ, which releases tuberin (TSC2) and phosphatidylinositol 3-kinase catalytic subunit type 3 (VPS34). Therefore, ENDOG can trigger autophagy by inhibiting one of the most important pathways that negatively regulates autophagy—the mammalian target of the rapamycin (mTOR) pathway—[163].

To prevent ROS from causing irreparable damage, the mitochondria resort to an ample set of enzymes. Mitochondrial ROS-specific sensors can initiate retrograde signalling. Further, lipid-, e.g., cardiolipin-oxidation products are well-known signalling molecules [165]. In addition, ROS sensing may also rely on proteins that are switchable by redox reactions. Cysteine is one of the amino acids that is most prone to oxidation. The mild oxidation of its thiol group to its sulfenic species by H_2_O_2_ is normally reversible. This PTM can be regulated specifically, as it is well-suited to modulate enzyme activity. It is effective in modulating the activity of TCA enzymes and ETC components [166]. However—to the best of our knowledge—cues about this PTM driving mitochondrial proteins outside the organelle are missing. This may reflect the kinetic rather than thermodynamic control of this PTM which makes the species populations highly dependent on the environment. 

The signalling of ROS based on reversible disulphide-bond formation between cysteine residues is ubiquitous [127]. Mammalian cells display a variety of redox sensors, but those triggering cell responses (e.g., KEAP1, FOXO, NADPH oxidase 4 (NOX4), etc.) exert all their roles outside the mitochondria and are beyond the scope of this text. However, methionine and cysteine oxidation may also play key roles at the mitochondrial import level [167,168,169]. 

Within mitochondria, ROS signalling additionally relies on the interplay between the demethoxyubiquinone Q9 hydroxylase—CoQ7 (a.k.a. MCLK-1) in yeasts and mammals, or CLK-1 in *C. elegans*—and its import system. The CoQ7 is an iron-dependent hydroxylase acting on the route of CoQ biosynthesis [170]. Furthermore, CoQ (a.k.a., ubiquinone) is a liposoluble molecule that carries electrons from complexes I and II, the electron-transferring-flavoprotein dehydrogenase (ETFDH), the glycerol-3P dehydrogenase (GPDH), and the dehydro-ororate dehydrogenase (DHODH) to complex III; complex I and complex III are the major ROS generators in the ETC. In addition, CoQ is reduced by other enzymes. Alterations in its biosynthesis—including mutations in CoQ7—cause a wide range of pathogenic phenotypes [170]. Notably, CoQ7 is highly conserved, and strong associations of CoQ7 with IMM-bound CoQ9 form a quaternary complex, together with additional proteins acting on the route [171]. The resulting complex alters IMM locally. This enables CoQ7 to bind the hydrophobic substrate, with its quinone ring properly fitting the catalytic site. In addition, CoQ7 is imported to the mitochondrial matrix in a ΔΨ-independent, heat hock protein 70 (HSP70)-dependent manner [172]. In yeasts, ETC activity drops to levels at which respiration cannot be sustained upon CoQ7 truncation or deletion [173]. The CoQ7 *C. elegans* homolog (CLK-1) can bind to the replication-origin OL region of mitochondrial DNA, depending on ADP levels, and mutations affecting this interaction extend the lifespans of worms [174]. Mutations in CLK-1 can also affect the mtDNA copy number; 2,4-dihydrobenzoate—a chemical used to rescue OXPHOS upon CoQ deficit—is unable to reverse his effect [175]. Thus, CLK-1 may play a role in the coupling of OXPHOS performance to mtDNA replication via ADP levels. 

In addition to its role in the mitochondria, CoQ7/CLK-1 also plays a role in cell nuclei. Indeed, its N-terminal sequence contains a nuclear targeting sequence that is cleaved upon mitochondrial import [172]. Furthermore, CoQ7 co-localises in the mitochondria and cell nuclei; the population size of nuclear CoQ7 is correlated with ROS levels [176]. In addition, CoQ7 can bind chromatin, according to ChIP experiments, in the WW domain-containing oxidoreductase (WWOX) and TIMM22 genes. Nuclear CoQ7 modulates the transcription levels of genes related to ROS metabolism. It increases those of glutaminase (CLNA-1/GLS2) while damping the expression of WWOX and genes related with the UPRmt, such as HSPA9, HSPD1, and AFG3L2, among others [176].

Defects in the mitochondrial import system physically trigger CoQ7-location changes upon oxidative stress, rather than CoQ7 PTM. As summarised for phosphosite [177], the human CoQ7 NTS—which is cleaved with the MTS when the protein enters the mitochondria—contains two residues (Ser22 and Tyr 26) whose phosphorylation takes place under ischemic stress [178]. The yeast homolog is also phosphorylated. Notably, ischemic tumours often show mutations of these residues [178]. Commercial protein kinase A (PKA) and C (PKC) can phosphorylate Ser22. In turn, protein phosphatase PPTC7—PTC7 in yeasts, belonging to the protein phosphatase 2C (PP2C) family—controls the phosphorylation of most of the enzymes involved in CoQ synthesis [179]. However, this enzyme acts within the mitochondrial matrix, where the cleaved, NTS-depleted-form of CoQ7 is located [180]. This suggests that oxidative stress impairs CoQ7 import, instead of re-directing the mitochondrial species to the nuclei. Nevertheless, the phosphorylation state of the nuclear species remains unknown. 

As in responses to hypoxia, there are multiple links between oxidative stress and the UPR. Indeed, the mitochondrial import system is sensitive to oxidative stress [167,168]. Further—as with SOD2—H_2_O_2_ levels can regulate protein import via HSP70’s dephosphorylation by PP2C [181]. Thus, the impairment of or dysfunction in mitochondrial import leads to the accumulation of unprocessed mitochondrial proteins in cytosol. Proteins such as SOD2, ATF5, ENDOG, and CoQ7 perform alternative functions outside the mitochondria; the other proteins remain unfolded, and they are redirected to the proteasome or cause stress. 

The recent improvements in gene-sequencing capabilities and bioinformatics have enabled the detection of short open-reading frames (sORFs) that may encode for small peptides in both nuclear and mitochondrial DNA. It is likely that most of these sORFs have a probabilistic origin, and that they are the result of the aleatory arrangement of DNA bases and, therefore, that they show low levels of evolutive conservation. If expressed, many of these sORFs may lead to unfunctional peptides, so a functional analysis at the peptide level and validation tools are needed [182]. On the other hand, the existence of some sORFs in the mtDNA could explain why the pathological effect of base substitutions does not affect the function of the canonical gene affected [183]. The mtDNA comprises a few sORFs encoding small factors, some of which are capable of modulating oxidative stress and protecting cells. The first, humanin (HN), is a 24-amino-acid peptide found during a screening of cDNA libraries for the rescue of Jurkat cells transformed with an amyloid-encoding plasmid [184]. These cells secreted HN, which protected them from antibody-peptide toxicity. More recently, a hexadecapeptide encoded within the 12S rRNA—the mitochondrial ORF of the 12S rRNA-*c* (MOTS-*c*)—was shown to promote homeostasis while decreasing obesity and insulin resistance [185]. Accordingly, the expression of this small gene is downregulated during the development of type II diabetes [186]. The MOTS-*c* transcript comprises a strong eukaryotic translation-initiation sequence, and it is encoded for eukaryotic translation, the product synthesis taking place in the cytoplasm (Figure 2) [185]. However, immunofluorescence and cell-fractionation assays show that it is located in mitochondria during homeostasis, but, as with HN, it can be found in plasma; therefore, it also acts as a hormone [185]. The peptide modulates the performance of purine synthesis, acylcarnitine metabolism, and the methionine cycle. Its ability to inhibit purine synthesis, in turn, activates AMPK, thereby affecting lipid and glucose metabolism. Metabolic or oxidative stress causes the AMPK-dependent translocation of MOTS-*c* to the cell nucleus [187]. Notably, the peptide lacks a complete NLS, and only a stretch of hydrophobic residues is needed for translocation, suggesting that it is mediated by interactions with another protein. The MOTS-c binds to the DNA in the nucleus, specifically aiming at the antioxidant response elements (ARE) sequences (5′-TGACnnnGC-3′) of target genes. The most prominent of these targeted genes are those encoding heme-oxygenase-1 (HO-1), ferritin, and NAD(P)H dehydrogenase quinone 1 (NQO1), key players in the control of oxidative stress and protein ubiquitination. Further, MOTS-c also interacts with the activating transcription factors 1 and 7 (ATF1/ATF7), as well as with the nuclear factor erythroid-2 related factor 2 (NRF2). Both of these also target AREs, in addition to other promoter elements [188,189]. Thus, MOTS-c acts synergically with the cytoplasmic stress-response routes, including the p62-KEAP1-NRF2 axis, which is also known to be activated by ROS and ROS-sensitive kinases. In addition, MOTS-*c* directs the expression of cytokine genes, downregulating the expression of proinflammatory genes while increasing anti-inflammatory IL-10, helping to restore their balance under pathological conditions. 

### 3.3. The Mitochondrial Unfolded-Protein Stress Response Targets Nuclear Genes

Except for those that are intrinsically disordered, proteins need to be properly folded in the cell compartment to exert their function. Proteostasis, the driving governance of proteome functionality, relies on quality control and stress-adaptation mechanisms, such as the heat-shock response. Unfolded protein responses occur in a variety of organelles, including the ER and mitochondria. Given the concentration of ROS within the organelle, this regulation ensures the correct performance of its activity. 

The UPR^mt^ drives the transcriptional activation of nuclear genes encoding chaperone proteins and proteases to maintain mitochondrial proteostasis [190,191]. Dysfunctional UPR^mt^ is related to pathologies such as metabolic syndrome, fatty liver, and diabetes. A variety of stressors affecting the mitochondrial machinery—including ROS, protein aggregation, the failure of the protein-quality-control system, or the deadening of the ETC—can trigger UPR^mt^.

In *C. elegans*, nuclear-encoded mitochondrial-stress-related activating transcription factor 1 (ATFS-1) is a key factor in UPR^mt^. This ATFS-1, which is encoded by the ZC376.7 gene, is a basic-region leucine-zipper (bZIP) protein homologous to mammalian ATF5. When the amount of misfolded proteins within the matrix overwhelms the restoring chaperone activity, the serine AAA^+^ protease CLPP-1 digests them, yielding a variety of small peptides [192]. Next, an ATP binding cassette (ABC) transporter, HAF-1, transports them to the IMM, from where they and the proteolytic products of YME1 activity within this space diffuse to cytosol through porin or the TOM complex or, alternatively, a second ABC transporter exports them to cytosol [193]. Again, the interaction of these peptides with the mitochondrial import system is purported to prevent ATFS-1 entry into the organelle. As ATFS-1 presents an NLS motif, the impairment of mitochondrial import drives this protein to the cell nucleus. In this compartment, it binds to A-rich promoter elements known as UPR^mt^ elements (UPR^mt^E), driving the transcription of genes encoding mitochondria-related protein chaperones, HSP60 and HSP70, as well as others governing glycolysis/gluconeogenesis, ribosome synthesis, TCA enzymes, OXPHOS proteins, and autophagy regulators [194].

In mammals, a deletion mutant of the mitochondrial ornithine transcarbamilase (OTC), which is prone to aggregation, was shown to trigger an integrated stress response at the cellular level [195]. The latter involved the activation of genes encoding the C/EBP homology protein (CHOP)—a transcription factor that is known to be involved in the response to a variety of cellular stresses, together with C/EBPb—and the AAA+ protease LON, instead of the CLPP-1 homolog, which is expendable in relation to UPR^mt^ initiation in mammals [196]. Recent independent works have unveiled a pathway through which different mitochondrial dysfunctions lead to the phosphorylation of the eukaryotic initiation factor 2, subunit I (eIF2α) by the heme-regulated inhibitor (HRI) [197,198]. Under stress, the OMA1 protease processes the DAP3-binding cell-death enhancer 1 (DELE1) to a truncated form (sDELE1) lacking its N-terminal region, which is then released to cytosol. Next, sDELE1 interacts with HRI through its C-terminal tricopeptide repeats, activating its kinase activity. Phosphorylated eIF2α then sparks the translation of CHOP, ATF4, and ATF5 mRNAs, leading to an integrated stress response [199]. Additionally, a member of the ATF/CREB family (ATF5, a.k.a. ATFX) was shown to aid cell survival by increasing the transcription of antiapoptotic genes [200]. In addition, CHOP triggers the expression of ATF4 and ATF5 [201]. Furthermore, ATF5, the mammal ortholog of ATFS1, also holds an NLS motif, as well as a sorting sequence weakly targeting the protein to the mitochondria, in addition to is bZip domain. The ATF5 can complement ATFS-1 *C. elegans* KO, binding to UPR^mt^E-containing promoters [199]. Thus, ATF5 supports cell proliferation and helps to restore mitochondrial activity under stress. 

The crosstalk between general stress-response pathways and the UPR^mt^ exceeds the role of CHOP as a trigger. Overall, proteotoxic stress at the cellular level also affects the mitochondria; therefore, it may elicit a response. Upon heat shock, mitochondria crowd around the perinuclear space. Next, the formation of the mitochondrial permeability transition pore (MPTP) facilitates the translocation of the mitochondrial single-strand binding protein 1 (mtSSBP-1) to the cell nucleus [202]. The mtSSBP-1 belongs to the oligonucleotide/oligosaccharide binding (OB)-fold protein family. These OB-fold proteins contain small b-barrel domains, capped by an α-helix, with variable loops, which are strongly related to their specific functions [203]. Under homeostatic conditions, mtSSBP-1 takes part in the mtDNA-replication machinery [204]. Upon MPTP opening, mtSSBP-1—which lacks NLS—binds heat-shock factor 1 (HSF-1) through its trimerization domain in the cytoplasm and, subsequently, both proteins are directed to the nucleus. The complex binds other co-activators, such as Brahma-related gene-1 (BRG1), and triggers the transcription of diverse chaperones and other response factors [202,205].

### 3.4. Mitochondrial Factors in the Response to Nuclear DNA Damage

Damage to DNA occurs continuously in cells under different exogenous stresses (e.g., UV light, ionizing radiation, and exposure to chemical attack by electrophiles or free radicals) and endogenous threats (e.g., replication errors, DNA-base mismatches, topoisomerase–DNA complexes, spontaneous base deamination, cellular metabolism, and oxidative stress), which can ultimately lead to single- or double-strand DNA breaks [206,207]. Tens of thousands of such DNA-damage events occur every day in our cells [208]. The most common DNA lesions are hydrolytic cleavages of the glycosidic bond between the DNA base and the sugar-phosphate group—leading to base-less sites—and the hydrolytic deamination of the DNA bases [209]. Furthermore, the oxidation, nitrosylation, or alkylation of nitrogenous bases can be generated by normal metabolism products, whereas breaks in the phosphate-deoxyribose backbone can arise in response to high levels of energy radiation or during DNA replication [210]. Such DNA lesions can adversely affect DNA replication and transcription, leading to replication-fork collapse or transcription arrest, or serving as mutagenic templates [211]. Consequently, if DNA modifications persist, they lead to mutations or genome aberrations that threaten cell or organism viability. 

To deal with this threat, life has evolved many different mechanisms to detect, signal, and repair DNA injuries, which are collectively termed the DNA-damage response (DDR) [212,213]. At least five major DDR mechanisms—non-homologous-end-joining (NHEJ), homologous recombination (HR), mismatch repair (MMR), nucleotide excision (NER), and base excision (BER) repair—are involved in damage sensing, the blocking of the cell-division cycle, providing accurate DNA repair, and promoting abnormal cell apoptosis [214,215]. Not surprisingly, defects in the DDR mechanisms are often associated with carcinogenesis, apoptosis, and senescence [211]. Some of the most hazardous forms of DNA injury are DNA double-strand breaks (DSBs), which can be repaired by HR or NHEJ, depending on availability of a homologous DNA sequence for use as a template [216]. The HR appears to be the predominant mechanism of DSB repair in yeast, and it is restricted to the S and G2 phases of the cell cycle—when a sister chromatid is available. By contrast, NHEJ can operate in all cell-cycle phases and seems to be the major pathway for the repair of DSBs in mammals [217]. MMR removes mis-paired nucleotides, as well as the insertion or deletion loops formed during replication or recombination [218]. Furthermore, NER is the pathway responsible for repairing the so-called pyrimidine dimers, while the BER pathway corrects damage resulting from the oxidation, methylation, or deamination of bases.

**Figure 3:**
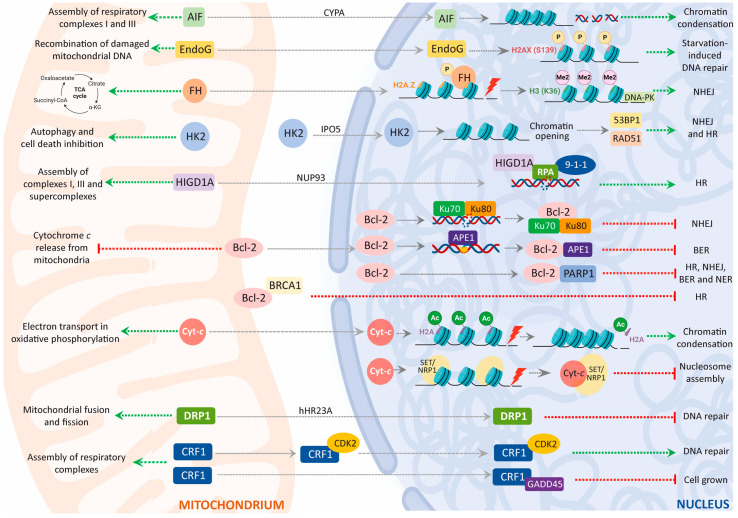
Protein trafficking from mitochondria to nucleus during DNA damage. Mitochondrial proteins can be targeted to the cell nucleus to modulate the chromatin dynamics and/or take part in DNA repair. In the nucleus, apoptosis-induction factor (AIF) promotes chromatin condensation and DNA fragmentation, while endonuclease G (ENDOG) increases g-H2AX levels and favours DNA repair. AIF translocation to the nucleus is mediated by cyclophilin A (CYPA). The metabolism-related enzymes fumarate hydratase (FH) and hexokinase 2 (HK2) can be located in the nucleus, where FH promotes dimethylation of histone H3 to recruit DNA-dependent protein kinase (DNA-PK), thereby enhancing non-homologous end-joining (NHEJ), whereas HK2 favours chromatin opening, which results in increased expression levels of p53 binding protein 1 (53BP1) and RAD51 recombinase, promoting NHEJ and homologous recombination (HR), respectively. Nuclear translocation of HK2 is exerted by importin IPO5. Hypoxia-inducible gene-domain-family member 1A (HIGD1A) moves into the nucleus in response to double-strand breaks (DSBs) with the help of nucleoporin NUP93. Next, nuclear HIGD1A recruits the replication protein A (RPA) and the 9-1-1 complex, thereby prompting HR. B-cell lymphoma 2 (BCL-2) protein accumulates in the nucleus and interacts with the Ku70-Ku80 heterodimer to inhibit NHEJ. Nuclear BCL-2 also docks apurinic/apyrimidinic endonuclease (APE1)—hampering base excision repair (BER)—as well as poly (ADP-ribose) polymerase-1 (PARP-1), thereby abolishing NHEJ, HR BER, and nucleotide excision repair (NER) pathways. BCL-2 and breast cancer tumour suppressor 1 (BRCA1) co-localise to mitochondria, resulting in the decrease in nuclear BRCA1, and subsequently inhibiting HR. Cytochrome *c* (Cyt-*c*) migrates to the cell nucleus following DSBs, where it displaces the acetylated histone H2A from the chromatin, thereby producing chromatin condensation. Furthermore, nuclear Cyt-*c* interacts with suppressor of variegation, enhancer of zeste, and Trithorax (SET), or nucleosome assembly protein 1 (NAP1)-related protein 1 (NRP1), inhibiting their nucleosome-assembly abilities. Dynamin-related protein 1 (DRP1) is translocated into the nucleus by human homologue of yeast Rad23 protein A (hHR23A), where it hinders DNA-damage repair. Under ionizing radiation, CR6-interacting factor 1 (CRIF1) moves to the nucleus, where it mediates the nuclear transport of cyclin-dependent kinase 2 (CDK2) to help with DNA-damage repair. Moreover, nuclear CRF1 negatively regulates cell growth through its interaction with growth arrest and DNA-damage-inducible protein 45 (GADD45). TCA: tricarboxylic acid cycle.

Nuclear DNA damage is an important initiator of mitochondria–nucleus crosstalk, which is related to age-associated diseases [219]. In fact, several DDR-deficient ageing disorders—such as Cockayne syndrome, xeroderma pigmentosum group A (XPA), and ataxia telangiectasia—comprise mitochondrial alterations consisting of increased mitochondrial membrane potential and increased oxygen-consumption rates [220,221,222]. The question of how DNA damage appeals to mitochondria signalling is a new area of investigation [219]. The mitochondria-to-nucleus crosstalk is clear because of the presence of mitochondrial proteins in the latter organelle (Figure 3). Indeed, various metabolism-associated proteins do translocate to the nucleus under DNA damage. We review this process below.

#### 3.4.1. AIF and DNA damage

Treatment with DNA-damage inducers, such as doxorubicin, actinomycin D, or topoisomerase I inhibitor camptothecin, causes AIF translocation to the nucleus [142]. Furthermore DNA-damage-induced _t_AIF translocation and apoptosis have been shown to depend on the presence of p53 and a pro-apoptotic member of the B-cell lymphoma-2 (BCL-2) family, BCL-2-associated X protein (BAX) [142,151]. The translocation of _t_AIF to the nucleus is apparently controlled by CYPA and heat-shock protein 70 (HSP70) through the direct binding of these two proteins to _t_AIF [223]. In addition, CYPA is a positive regulator of the cytosolic-to-nuclear redistribution of _t_AIF [153], whereas HSP70 blocks this translocation, thereby suppressing _t_AIF-mediated apoptosis [224]. Remarkably, the nuclear accumulation of _t_AIF is hampered by the expression of the E3 ubiquitin ligase parkin—a protein that promotes dopaminergic neuron survival—in cells exposed to apoptogenic treatment, which contributes to the neuroprotective activity of parkin [225]. 

#### 3.4.2. ENDOG

As pointed out above (Section 3.2), ENDOG is also involved in DDR [163]. Furthermore, ENDOG elicits DDR in starvation conditions to promote autophagy [163]. Damage to DNA is an early event following starvation-induced autophagy, and it is mediated by poly-(ADP-ribose) polymerase-1 (PARP1) and AMP-activated protein kinase (AMPK) [226]. The loss of ENDOG blocks the activation of PARP1 and AMPK, thus repressing starvation-induced DNA damage [163]. The latter observation proves that ENDOG-mediated DNA-damage response promotes autophagy. In fact, ENDOG without endonuclease activity does not induce the DNA-damage response or autophagy [163]. Cell treatment with the DNA-damage inducer etoposide—a DNA topoisomerase II inhibitor—enhances the ENDOG-induced DDR (measured as increments in the DNA-damage sensors γ-H2AX and phosphorylated ataxia-telangiectasia mutated or p-ATM), and also increases autophagosome formation [163]. Furthermore, mitochondrial ENDOG is released and translocated to nuclei to sustain nuclear DNA damage in autophagy-defective cells. This suggests that DNA-damage-induced autophagy is not needed for recovery from nuclear DNA damage [227]. Although DNA damage induces ENDOG’s nuclear translocation, the mechanism underlying this translocation and the specific role of ENDOG in DNA repair are still under investigation.

#### 3.4.3. Fumarase

In response to DSBs, yeast fumarase is recruited from cytosol to the nucleus, where it seems to play a key role in the protection of cells from DNA damage [228]. The enzyme fumarase, or fumarate hydratase (FH), takes part in the tricarboxylic acid (TCA) cycle in the mitochondrial matrix. It catalyses the reversible conversion of fumaric acid to malic acid. Fumarase redistribution between the mitochondria and cytosol has been observed in all the eukaryotes studied, including yeast and humans [228]. In yeast, it has been proposed, cytosolic fumarase participates in the catabolism of amino acids [229], and as a scavenger of fumarate from the urea cycle [230]. Nevertheless, this does not explain the high levels of FH in cytosol [228]. Apparently, fumarase is first targeted to mitochondria, but a sub-population of the enzyme is located in cytosol [231]. If, during import, the nascent chain begins to fold in the mitochondrial matrix, it is localised in the mitochondria, but if the import slows and the polypeptide chain begins to fold in the cytosol, the chain withdraws from the import machinery, and it is localised in the cytosol [232].

Upon the induction of DSBs, a lack of nuclear fumarase prevents histone H2AX phosphorylation and cell-cycle-checkpoint activation, making yeast and human cells more sensitive to DNA damage. This suggests that fumarase is related to the early detection or signalling of DSBs. This protective nuclear effect depends on the enzymatic production of fumaric acid by fumarase. High concentrations of this metabolite can indeed overcome the absence of the enzyme [228]. To perform its DDR-related role, FH does not accumulate at the sites of breaks in separate nuclear foci, as is the case with many members of the DDR. 

Recent evidence shows that nuclear fumarase promotes DNA repair in response to ionizing irradiation through the hindering of histone demethylation [233,234]. Thus, exposure to ionizing radiation induces the phosphorylation of nuclear fumarase—at Thr 236—leading to an interaction between fumarase and the histone variant, H2A.Z, adjacent to DSB regions [233]. This FH recruitment results in the local accumulation of fumarate in DSBs, which inhibits the activity of lysine-specific demethylase 2B (KDM2B). This results in the enhanced dimethylation of histone H3—at Lys 36—which, in turn, favours the accumulation of DNA-dependent protein kinase (DNA-PK) at DSB foci [233]. In NHEJ, DSBs are recognised by the DNA–PK complex—consisting of the heterodimer, Ku70-Ku80, and the catalytic subunit, DNA-PKcs—which then recruits end-processing enzymes, polymerases, and DNA ligase IV [208]. Altogether, this evidence proves that nuclear fumarase actively participates in DNA repair by NHEJ in mammalian cells. 

On the other hand, a tumour-suppressing role has also been proposed for fumarase in human cells, owing to FH mutations, which predispose individuals to common types of tumour [235]. Accordingly, FH inhibition leads to elevated intracellular fumarate, which in turn acts as a competitive inhibitor of hypoxia-inducible factor (HIF) prolyl hydroxylase, further stabilizing HIF by preventing its proteasomal degradation [236]. Subsequently, the transcription factor HIF promotes tumour growth and survival by inducing glycolysis and blood-vessel growth—facilitating the adaptation of tumoral cells to low-oxygen conditions [237]. However, in some cases of FH deficiency, there is no accumulation of fumarate, nor is there stabilization of HIF [238]. The latter observation can be explained by the above-mentioned role of fumarase in the DDR, suggesting that the tumorigenesis function of FH is due to its role in the cellular response to DSBs. Finally, high fumarate levels in FH-defective cells have been shown to increase genetic instability by impairing cell-cycle G2 arrest and causing early mitotic start in response to DNA damage [239]. 

#### 3.4.4. Hexokinase 2

Hexokinase 2 (HK2)—another mitochondrial enzyme—has been reported to modulate the DDR. Hexokinases are a family of ubiquitous enzymes, primary found in cytosol, which participates in the first and rate-limiting step in glycolysis by catalysing the phosphorylation of glucose to glucose 6-phosphate. Hexokinase 2 is the second most predominant isozyme in the family, and it is mainly expressed in insulin-sensitive tissues—the heart, skeletal muscles, adipose tissues, and a wide range of tumours [240]. It can dock to mitochondria, where it performs additional functions in autophagy and cell-death inhibition, which are independent of its enzymatic activity [241]. Mitochondrial HK2 contributes to the metabolic reprogramming of tumoral cells by enhancing glucose consumption and lactate production, as well as decreasing mitochondrial respiration in parallel [241]. Interestingly, the anchoring of HK2 to the outer mitochondrial membrane is key to its oncogenic activity, and it is regulated by SUMOylation. 

Furthermore, HK2 can localise to the nuclei of acute myeloid leukaemia (AML) and normal hematopoietic stem cells, and it is involved in the maintenance of stemness [242]. Importantly, nuclear HK2 alters stem-cell function and differentiation independently of its enzymatic function. The nuclear import of HK2 is mediated by importin IPO5, whereas HK2 nuclear export requires exportin 1 (XPO1) [242]. In addition, HK2 can localise to the nuclei of acute myeloid leukaemia (AML) and normal hematopoietic stem cells, and it is involved in the maintenance of the stemness [242]. 

Remarkably, nuclear HK2 interacts with several proteins associated with the DDR pathways in stem cells. Accordingly, the overexpression of nuclear HK2 in stem cells treated with a chemotherapeutic agent that causes DSBs, daunorubicin, results in a decrease in the number of DSBs—measured by the levels of the DSBs marker g-H2AX—and in increased expression levels of tumour-suppressor p53 binding protein 1 (53BP1) and RAD51 recombinase, which are essential factors for NHEJ and HR, respectively [242]. Thus, nuclear HK2 seems to mediate the enhanced DNA-damage responses of AML stem cells. 

On the other hand, nuclear HK2 modulates chromatin accessibility and maintains DNA integrity by interacting with proteins involved in chromatin organisation and regulation, such as the RNA-polymerase-associated protein CTR9, myc-associated factor X (MAX), the histone lysine demethylase plant homeodomain finger (PHF) protein 8 (PHF8), protein 10 (PHF10), and spindlin1 (SPIN1) [242]. In addition, MAX recognises basic helix–loop–helix (bHLH) E-box motifs, which are implicated in cell proliferation, stem-cell maintenance and differentiation, and the DNA-damage response [243]. The overexpression of nuclear HK2 increases chromatin accessibility, whereas its downregulation decreases chromatin accessibility, demonstrating that stem cells have more accessible chromatin [242]. It can be inferred that the opening of the chromatin structure enhances DNA-damage repair by increasing access to DNA-repair proteins. Additionally, nuclear HK2 also interacts with proteins related to the DNA-damage response, e.g., the NAD-dependent protein deacetylase Sirtuin 1 (Sirt1), tyrosyl-DNA phosphodiesterase 2 (TDP2), and ubiquitin-protein ligase N-recognin 5 (UBR5, a regulator of transcription in damaged chromatin) [242].

#### 3.4.5. HIGD1A and DNA Damage

In cells, responses often result from crosstalk between pathways that are initially unconnected. Thus, HIGD1A was found to be expressed during hypoxia. Some metabolic stress—e.g., glucose starvation coupled with hypoxia or DNA damage induced by etoposide—triggers its nuclear accumulation, as well as its interaction with nuclear AIF [244]. A more recent study found that HIGD1A moves into the nucleus in response to DSBs, thereby participating in the regulation of the HR pathway of DDR by interacting with replication protein A (RPA) [245]. Consequently, RPA binds and stabilises single-stranded DNA intermediates, thus playing an essential role both in DNA replication and in the cellular response to DNA damage [246]. In the early stages of HR, nuclear HIGD1A promotes RPA loading at DSB sites and enhances the chromatin binding of the 9-1-1 complex, thereby activating the ataxia–telangiectasia and Rad3-related (ATR)-mediated phosphorylation of checkpoint kinase 1 (CHK1)—a G2/M-cycle-damage checkpoint, resulting in a high survival rate [245]. By contrast, in the later stages of HR and after promoting RPA binding, HIGD1A inhibits the aberrant persistence of RPA by enhancing RPA ubiquitination and its subsequent proteasomal degradation, which enables the accomplishment of HR [245]. Furthermore, the absence of nuclear HIGD1A has been linked to the impairment of the DDR displayed by replication stress in cells deficient in COX4-1 (a common isoform of COX subunit 4) [247]. 

The mechanism underlying the nuclear translocation of HIGD1A observed in response to ionizing radiation involves the recognition of HIGD1A by the nucleoporin NUP93 through a conserved sequence (residues 46 to 60) of HIGD1A [245]. Intriguingly, the nuclear localization of HIGD1A is also dependent on the presence of BCL-2-related proteins, specifically BAX and BCL-2 homologous antagonist/killer (BAK), positive regulators of the intrinsic pathway of apoptosis, which suggests that HIGD1A requires apoptosis-related molecules to be translocated into the nucleus [244,248].

#### 3.4.6. B-Cell Lymphoma 2 Proteins and DDR

The members of the BCL-2 family act on DDR by means beyond the modulation of the translocation of mitochondrial factors. The BCL-2 family of proteins consists of 25 pro- and anti-apoptotic members that maintain a balance between newly forming cells and old dying cells [249]. The members of the family localise to endomembranes (of the mitochondria and the endoplasmic reticulum (ER)) through a transmembrane (TM) domain and govern the permeability of transition pores by interacting with each other [249,250]. The BCL-2 family members share their homology in the BCL-2 homology (BH) domains [251]. When anti-apoptotic members are overexpressed, the ratio of pro- and anti-apoptotic BCL-2 family members is disturbed, and apoptosis is avoided [252]. Consequently, the targeting of the anti-apoptotic members of the BCL-2 family triggers apoptosis and, thus, overcomes drug resistance to cancer chemotherapy [253]. In addition to their accepted role in apoptosis regulation, several studies suggest roles for BCL-2 members in DNA repair, specifically for the BCL-2 protein.

The BCL-2 protein is an anti-apoptotic factor anchored to the external mitochondrial membrane, the ER, as well as in the outer nuclear envelope [254]. Pro-survival members of the BCL-2 family—including BCL-2—possess four BH domains, i.e., BH1, BH2, BH3, and BH4 [251]. The BCL-2 protein performs its anti-apoptotic activity by impairing the release of Cyt-*c* from the mitochondrial IMS, thus preventing caspase activation [252]. This anti-apoptogenic activity makes the BCL-2 protein behave as an oncogene and a potent tumour promoter [255]. 

Some studies highlight the links between BCL-2-dependent carcinogenesis and genetic instability due to impaired DNA repair [256,257]. Indeed, in addition to anti-apoptotic activity, BCL-2 negatively regulates the NHEJ pathway. The BCL-2 protein suppresses DSB repair by inhibiting the heterodimer, Ku70-Ku80—part of the DNA-PK complex functioning in NHEJ—and, thus, increasing genetic instability [258]. The Ku70-Ku80 heterodimer recognises and binds to DSBs ends. Subsequently, the catalytic subunit (DNA-PKcs) joins to form the active DNA–PK complex, which recruits end-processing enzymes and other factors to DSBs [259,260]. Ionizing radiation stimulates the accumulation of BCL-2 in the nucleus, which then interacts with the Ku70-Ku80 heterodimer and inhibits its binding to DSB ends [258]. Remarkably, BCL-2 interacts with Ku70 and Ku80 via its BH1 and BH4 domains, which are the regions responsible for BCL-2 nuclear accumulation in response to ionizing radiation [258]. It is unclear whether such effects of BCL-2 in the nucleus are caused by an increase in its expression or by its translocation from the mitochondria to the nucleus following DNA damage.

The BCL-2 protein also regulates DDR by interacting with breast cancer tumour suppressor 1 (BRCA1), which plays an essential role in HR [261]. The BCL-2 protein and BRCA1 co-localise to mitochondria and the ER through the TM domain of BCL-2, leading to a decrease in nuclear BRCA1 and, subsequently, the inhibition of HR [261]. Importantly, targeting BRCA1 through BCL-2 not only affects HR, but also other DDR pathways, such as NHEJ or BER, as well as several cellular processes, e.g., cell-cycle regulation, cell death, or the inactivation of the X-chromosome [250]. 

Furthermore, BCL-2 attenuates DNA repair by the NER and BER pathways. It has been reported that BCL-2 avoids the NER-mediated restoration of cyclobutane pyrimidine dimers (CPD), kinds of photo-induced DNA lesion, although the underlying molecular mechanisms have not been explored [262]. On the other hand, BCL-2 inhibits the BER-dependent correction of the chemical changes produced in the DNA bases [257]. First, BER is started by the elimination of the damaged base to generate an abasic site (AP). Next, apurinic/apyrimidinic endonuclease (APE1) cleaves the DNA to the 5′ end of the AP site, thus creating a space for the filling with one nucleotide [250]. To become active, APE1 forms a complex with X-ray repair cross-complementing protein 1 (XRCC1) [250]. Scholars have proposed two mechanisms involving APE1 to explain the inhibition of BER exerted by BCL-2: (i) BCL-2 increases the expression of *c*-MYC, resulting in a decrease in the gene expression of APE1 [263]; and (ii) BCL-2 and APE1 establish a direct interaction, which destabilises the APE1–XRCC1 complex, resulting in the reduced endonuclease activity of APE1, thereby hampering BER [264].

Researchers have reported a direct interaction between BCL-2 and the enzyme PARP1 in the nuclei of B-cell lymphoma cells, which suppresses PARP1 enzymatic activity, thereby hampering DNA repair [265]. One of the earliest events in the DDR is the recruitment of PARP1 to diverse types of DNA lesion. The PARP1 is rapidly recruited to sites of DNA damage through its DNA-binding domains [266], where it catalyses the attachment of a negatively charged polymer—poly(ADP-ribose) or PAR—to itself and to multiple target proteins [267]. Subsequently, DDR-related proteins can be recruited to sites of DNA injury by binding to PAR through non-covalent interactions [268]. This PARylation activity not only contributes to the repair of single-strand breaks (SSBs) and DSBs—through HR and NHEJ—but also seems to be involved in BER and NER DNA-repair pathways [267]. Interestingly, BCL-2 is overexpressed and located within the nuclei of several tumour-cell lines [258,263,269], and then colocalises and inhibits PARP1 to attenuate DNA repair [265]. Lastly, it has been hypothesised that the inhibition of PARP1 by BCL-2 also suppresses a form of non-apoptotic cell death named parthanatos (from PARylation and θανατοζ, or death, in Greek) [265], distinct from apoptosis, necrosis or autophagy, in which PARP1 triggers the nuclear translocation of mitochondrial AIF and cell death [270], and then colocalises and inhibits PARP1 to attenuate DNA repair [265]. 

In addition, BCL-2 plays a role in the MMR pathway. When initiating MMR, a protein named mutS homolog 2 (MSH2) forms several complexes that recognise base–base mismatches and insertion/deletion mispairs [271]. Scholars have proposed two different mechanisms to explain the BCL-2 inhibition of MMR. According to the first, BCL-2 decreases MSH2 gene expression [256], whereas in the second, BCL-2 is translocated to the nucleus to sequester MSH2 through a direct interaction [269]. Both mechanisms reduce the effectiveness of MMR [250]. Intriguingly, the second mechanism is dependent on the BH4 domain of BCL-2, which is also needed for BCL-2 nuclear accumulation under genotoxic stresses [250].

#### 3.4.7. Cytochrome *c*

The Cyt-*c* is a heme-containing protein that resides in the intermembrane spaces of mitochondria as a component of oxidative phosphorylation, and transports electrons between cytochrome *bc*_1_ (complex III) and COX (complex IV) [272]. In mammalian cells undergoing apoptosis, Cyt-*c* is released to the cytosol, where it binds to the apoptosis-protease-activating factor 1 (APAF-1), as well as to procaspase-9, to form a huge macromolecular complex, the so-called apoptosome [12]. This results in the activation of caspase-9, which, in turn, triggers the activation of effector caspases—i.e., caspase-3 and caspase-7, which are responsible for the morphologic and biochemical changes associated with apoptosis, which execute cell death [12]. Certainly, Cyt-*c* is released from mitochondria in numerous organisms, including plants, yeasts, and flies, in addition to mammals [273,274,275]. 

Beyond the cell-death-related functions of Cyt-*c* in cytosol as a key element in the activation of mammalian caspases [12,276] or plant-caspase-like enzymes [277], numerous studies have shown that the heme protein migrates to the cell nucleus under non-apoptotic conditions [278,279,280,281,282,283]. Following DNA DSBs, Cyt-*c* gradually collects in the nucleus and displaces the acetylated histone H2A from the DNA, thereby producing the chromatin condensation and nuclear pyknosis observed during apoptosis [279]. However, the implications of such chromatin changes in the DNA-repair response have not been clarified.

When genotoxic stress leads to DNA breaks, Cyt-*c* shuttles to the nuclei of mammalian cells to interact with the suppressor of variegation, the enhancer of zeste, and trithorax (SET)/template-activating factor (TAF)-Iβ, a.k.a., SET/TAF-Iβ [281]. The SET/TAF-Iβ is a histone chaperone primarily located in the cell nucleus, where it mediates histone incorporation or the removal of nucleosomes during chromatin remodelling [284]. This chaperone is also recruited to the DSBs and, in fact, it is a key modulator of DNA repair in the chromatin surrounding those sites [285]. Accordingly, in response to the damage to DNA caused by the use of radiomimetic drugs, the downregulation of SET/TAF-Iβ increases DDR, and, by contrast, the upregulation of the chaperone impairs DNA repair. Under such conditions, SET/TAF-Iβ interacts with the Kruppel-associated box (KRAB)-associated co-repressor (KAP1), resulting in the retention of KAP1 and heterochromatin protein 1 (HP1) on chromatin, thereby hampering DNA repair by HR [285]. Therefore, SET/TAF-Iβ is recruited to DNA breaks to moderate uncontrolled DDR. In addition, upon the triggering of DNA breaks, nuclear Cyt-*c* binds to SET/TAF-Iβ and to its plant orthologue—nucleosome assembly protein 1 (NAP1)-related protein 1 (NRP1)—in mammalian and plant cells, respectively, thereby inhibiting their histone-chaperone abilities [281,286]. Although these observations suggest a role for nuclear Cyt-*c* in the DDR, further research is needed to clarify whether such Cyt-*c* -mediated chaperone inhibition results in increased or decreased DNA repair. 

In addition to the chromatin-remodelling and DDR-modulation activities of SET/TAF-Iβ, this chaperone also inhibits protein phosphatase 2A (PP2A) [287], which dephosphorylates a number of DDR effectors, including the phosphorylated variant of the histone H2AX (γ-H2AX) [288]. The γ-H2AX histone signals and recruits DDR factors on chromatin surrounding DSBs, so that γ-H2AX foci must be removed to proceed with the repair [288]. Using structural and computational approaches, it has been reported that Cyt-*c* forms a dynamic ensemble with SET/TAF-Iβ able to activate PP2A [289]. It is noteworthy that another histone chaperone and PP2A inhibitor—acidic leucine-rich nuclear phosphoprotein 32 family member B (ANP32B)—is also targeted by the haemoprotein in the cell nucleus upon DSBs, yielding Cyt-*c* -mediated PP2A activation.

In addition to SET/TAF-Iβ and ANP32B, Cyt-*c* forms complexes with other histone chaperones in the nucleus under DNA-damage conditions, as is the case of nucleolin (NCL) [290]. One of the most abundant proteins in the nucleolus of mammalian cells, NCL moves to the nucleoplasm after genotoxic stress, where it binds to histones H2A and H2B [291]. Under these conditions, NCL is recruited to DSB foci by binding to γ-H2AX [292]. Furthermore, this histone chaperone promotes cell-cycle arrest and DNA repair by binding to the mediator of DNA damage checkpoint protein 1 (MDC1) [292]. Moreover, NCL interacts with the DDR factors RAD50/51 and replication protein A (RPA) [291]. Remarkably, another abundant nucleolar protein, a histone chaperone named nucleophosmin (NPM), is specifically recognised by Cyt-*c* upon DNA breaks [293]. The metalloprotein binds to nucleolar NPM to drive the release of alternative reading frame (ARF)—a tumour-suppressor protein sequestered by NPM in the nucleoli [293]. In this way, mitochondrial Cyt-*c* may control the trafficking and availability of nucleolar proteins under stress conditions. Other histone chaperones recognised by Cyt-*c* in the cell nucleus are the heterogeneous ribonuclear proteins C1 and C2 (hnRNP C1/C2), which bind to damaged chromatin sites near DSBs [294]. In addition to chromatin remodelling, hnRNP C1/C2 participates in mRNA splicing, transport, stability, and translation [295], as well as in the NHEJ-repair pathway, by means of its interaction with the heterodimer Ku70-Ku80 [296].

#### 3.4.8. Dynamin-Related Protein 1

Dynamin-related protein 1 (DRP1) is a large GTPase belonging to the dynamin superfamily, and it is the key component of the mitochondrial and peroxisomal fission machinery [297]. Mitochondria constantly change their shape, keeping a balance between fusion and fission [298]. Mitochondrial fission requires the translocation of DRP1 from cytosol to the mitochondria, after which DRP1 presumably docks an adaptor protein on the outer membrane and uses the energy from GTP to self-assemble as helical- or spiral-shaped superstructures that constrict and cleave mitochondria [299,300]. Several studies link excessive mitochondrial fission and DRP1 with apoptosis, neuronal dysfunction, and cell death [301,302]. Recent investigations show that ionizing radiation triggers DRP1-dependent mitochondrial fission, and that DRP1 inhibition attenuates a form of cell death associated with aberrant mitosis—the so-called mitotic catastrophe [298].

Furthermore, DRP1-mediated mitochondrial fragmentation has also been associated with the cytotoxicity exerted by some commonly-used anticancer drugs—e.g., cisplatin. This links DRP1 with cancer progression and sensitivity to cancer treatments [303]. The expression of nuclear DRP1 is often found in lung adenocarcinomas, and it is correlated with poor prognoses, i.e., there is a significant difference in survival between patients with high levels of nuclear DRP1 and those with the cytosolic protein [303]. The nuclear transportation of DRP1 is mediated by its interaction with the human homologue of the yeast RAD23 protein A (hHR23A) [303]. In vitro, hypoxia raises nuclear DRP1 levels and cisplatin resistance in lung adenocarcinoma cells. How DRP1 accumulation in the nucleus causes resistance to antitumour drug treatments is still unknown. Conversely, DRP1 inhibition triggers replication stress, which then induces G2/M cell-cycle arrest and starts the DNA-damage response [304]. In addition, DRP1 loss causes centrosome overduplication and chromosomal instability. Thus, deficient mitochondrial fission—due to the loss of DRP1—causes genome instability [304]. Further, the disruption of DRP1 induces lethality and cellular senescence [305,306]. Thus, it is tempting to speculate that the genomic instability resulting from DRP1 deficiency is related to the ability of DRP1 to confer resistance to anticancer drugs. 

#### 3.4.9. CR6-Interacting Factor 1

The CR6-interacting factor 1 (CRIF1) is a mitochondrial protein involved in the assembly of the oxidative phosphorylation complexes into the inner mitochondrial membranes of mammalian cells [307]. The expression of CRIF1 expression is high in osteosarcoma tissues, and it has been associated with cancer radio-resistance [308]. 

In response to ionizing radiation, CRIF1 is translocated to the nucleus—it has a nuclear localization signal—where it negatively regulates cell-cycle progression and cell growth by means of its interaction with growth arrest and DNA-damage-inducible protein 45 (GADD45) [309]. A recent study states that CRIF1 prompts the nuclear transport of the DNA-damage-checkpoint regulator cyclin-dependent kinase 2 (CDK2) when DNA is damaged by ionizing irradiation [310]. Thus, CRIF1 promotes the phosphorylation of CDK2 at Thr14 and Thr160 in the cell nucleus, which ultimately facilitates G1/S-checkpoint activation and DNA-damage repair [310].

### 3.5. Mitochondrial Metabolites and Proteins Affecting Chromatin Remodelling

As pointed out in Section 2, mitochondrial functioning can affect chromatin remodelling to adapt response to changes in organelle performance, among other purposes. Thus, metabolites derived from and the activity of mitochondrial enzymes—including those in the TCA cycle—can influence cellular physiology through epigenetic modifications. In addition, diverse mitochondrial proteins can also take part in the governance of the chromatin state.

#### 3.5.1. Mitochondrial Metabolites and Chromatin Condensation State

##### α-Ketoglutarate, Succinate, and Fumarateple

Some mutations affecting enzymes in the TCA cycle, namely, isocitrate dehydrogenase, succinate dehydrogenase, or fumarase, leads to the accumulation of a-ketoglutarate, succinate, and fumarate in the mitochondria and cytosol [311,312]. 

The accumulation of α-KG leads to its conversion to 2-hydroxyglutarate, a potent inhibitor of α-KG-dependent dioxygenases. The most notable dioxygenases that are inhibited by excess α-KG are HIF prolyl hydroxylases [313] and the Jumonji-domain-family histone lysine demethylases (JMJ-KDMs) [314]. The inhibition of these dioxygenases leads to significant epigenetic and transcriptional changes [315]. Thus, the α-KG-mediated inhibition of HIF prolyl hydroxylases stabilises HIF, thereby activating hypoxic signalling, so 2-hydroxyglutarate is considered an oncometabolite, and it accumulates in many cancers [316]. It is believed that succinate accumulation has a similar impact on HIF prolyl hydroxylases, so this has also been linked to tumorigenesis [315,317]. Fumarate accumulation results in the succinylation of the cysteine residues of Kelch-like ECH associated protein 1 (KEAP1), which leads to the stabilization of the transcriptional factor nuclear factor erythroid-derived 2-like (NRF2), driving the activation of the NRF2 antioxidant transcriptional response [318]. Moreover, glutathione (GSH) can be succinylated, leading to the depletion of the cellular pools of GSH, which also results in activation of the NRF2-mediated antioxidant response [319]. Although it is seemingly beneficial, the persistent activation of the NRF2 response can be detrimental, and it has been linked to oncogenesis [319]. Strikingly, fumarate accumulation also results in increased trimethylation at Lys4 and acetylation at Lys27 of histone H3, processes that are related to the epigenetic changes observed in trained immunity [320]. Thus, fumarate acts as an inhibitor of the lysine demethylase 5 (KDM5) family of proteins responsible for H3K4 demethylation [321].

##### Flavin Adenine Dinucleotide

Flavin adenine dinucleotide (FAD) is a coenzyme derived from the vitamin riboflavin, which is produced in the mitochondria and acts as an electron carrier during catabolism. Importantly, FAD is also a cofactor for lysine demethylases [322]. Lysine-specific demethylase 1 (LSD1) is a flavin-containing protein endowed with FAD as a prosthetic factor [323]. Furthermore, LSD1 is a transcriptional corepressor, so it may catalyse the removal of methyl groups from lysine residues of histones whose methylation is linked to active transcription [322]. Remarkably, it is likely that alterations in the FAD/FADH_2_ ratio, which fluctuates with metabolic activities such as fatty-acid oxidation or the TCA cycle, affect LSD1-mediated demethylation [324].

#### 3.5.2. Mitochondrial Proteins and Chromatin Remodelling

Although most investigations are focused on mitochondrial metabolites as epigenetic regulators, some studies have also shown mitochondrial proteins that shuttle to the nucleus to control chromatin remodelling and, consequently, gene expression, through either their enzymatic or their transcriptional activities.

##### Mitochondrial Matrix Dehydrogenases

Pyruvate dehydrogenase (PDH) is an essential mitochondrial protein complex consisting of several copies of three enzymes, the pyruvate dehydrogenase (E1), dihydrolipoamide transacetylase (E2), and dihydrolipoamide dehydrogenase (E3) [325]. In addition, PDH is needed for connecting cytosolic glycolysis and the TCA cycle, through the oxidation of pyruvate to acetyl-CoA. 

In response to mitochondrial dysfunction—upon the inhibition of respiratory-chain complexes or the depletion of the mitochondrial outer-membrane protein, called mitochondrial carrier homolog 2 (MTCH2)—all three subunits of the PDH complex have been detected in cell nuclei, and it has been proposed that they shuttle there from mitochondria in a cell-cycle-dependent manner [326,327]. To the best of our knowledge, there are no clear data regarding the translocation mechanism of such a large complex. The nuclear PDH complex produces acetyl-CoA from pyruvate, leading to increased histone acetylation, which regulates the cell cycle. This is required for S-phase entry [326,327]. Remarkably, the nuclear-PDH-complex pool can also increase in response to growth signals, including the epidermal growth factor and serum, suggesting an important role in cell-cycle progression [326,327]. Recently, other mitochondrial matrix dehydrogenases have been shown to translocate during the reprogramming of somatic cells, as well as influencing histone acetylation [328]. Again, the translocation mechanisms are unknown. Thus, further research is needed to confirm this point.

##### Transcription Factor A

Mitochondrial transcription factor A (TFAM) is a nuclear-encoded member of a high-mobility group (HMG) family of proteins, which is essential for the transcription and replication of mitochondrial DNA [329]. It was recently reported that TFAM may also be located in the nucleus and, specifically, that it may be anchored to chromatin after the treatment of tumour cells with anticancer drugs [330]. This nuclear localization of TFAM is likely to be due to the presence of two nuclear localization signals within its two HMG domains [331]. However, the question of whether nuclear TFAM molecules are translocated from the mitochondria is a subject of debate. The TFAM binds tightly to the nuclear chromatin and regulates the expression of nuclear genes to promote cell growth [332]. The overexpression of TFAM enhances cell growth in several cancer-cell lines, while its downregulation inhibited their growth [332]. Thus, predictably, nuclear TFAM confers protection against the genotoxic drugs etoposide, camptothecin, and cisplatin [330]. Nuclear TFAM is also observed in neuronal cells, where the binding of nuclear TFAM to its promoter results in the suppression of its own expression [333].

##### Clock 1/Coenzyme Q7 Homolog

Clock 1 (CLK-1)—and its human homolog, coenzyme Q7 homolog (CoQ7)—is a mitochondrial enzyme involved in the biosynthesis of ubiquinone, a component of the electron transport chain [334]. In response to the mitochondrial accumulation of ROS, CLK-1/CoQ7 translocate to the nucleus to regulate gene expression in both *C. elegans* and human cells [176]. The nuclear fraction of CLK-1/CoQ7 binds to chromatin to regulate the gene transcription of the ROS-mediated response, triggering the adaptive oxidative stress response in order to decrease cellular ROS levels [176]. When CLK-1/CoQ7 are restricted to the nucleus, ubiquinone biosynthesis is inhibited, suggesting that CLK-1/CoQ7 exhibits different functions in the two compartments. 

The CLK-1 is an iron-binding protein endowed with hydroxylase activity [335]. However, it is unknown whether this activity is required for its nuclear role. In this regard, other iron-binding oxygenases have been shown to regulate gene expression through the demethylation of histones and cytosine bases [336].

## 4. Concluding Remarks

The key role of mitochondria in cell metabolism makes it a target for tight regulation and, conversely, a key governance agent for cells and tissues. This crosstalk between the control mechanisms evolved in the early stages and has grown in complexity over the course of evolution, to the point where it is fully integrated into major regulatory routes. In this work, we supplied an overview of the mechanisms involving mitochondrial proteins and soluble metabolites targeting cell nuclei, although we did not address the role of the plethora of mtRNA species that have recently emerged nor the complex signalling network involving the cytoplasm and other organelles, such as the ER and lysosomes. 

Several key features of the mitochondrial proteins targeting the nucleus are worth highlighting. Many of them may include an NLS element, enabling them to target the cell nucleus. Alternatively, they can also migrate by piggybacking on other proteins. Nevertheless, the mitochondrial import system is emerging as a key sorter of many of the regulatory proteins described here. The dependence of the import system on redox activity and its coupling to mitochondrial protein folding makes it an ideal mitochondrial-stress-signal transducer, in addition to membrane-pore transition.

The mitochondrial factors addressed in this work exert their actions in different ways and in a surprising variety of scenes, most often acting through moonlighting and in a pleiotropic manner. Moonlighting involves the participation of these proteins and metabolites in different processes. The pleiotropic manner involves the mitochondrial factors targeting a variety of genes in diverse ways, either directly or following a cascade of transcriptional events, post-translational modifications, or just shifting the accessibility of genes to processing machineries for repair or transcription. Under unbearable stress, some factors also aid DNA degradation. 

Finally, it is worth noting that the functional multiplicity of all these factors provides additional links between responses to different sources of stress, enabling them to either aid in the general stress responses of cells or elicit programmed cell death. We speculate that this may help to dampen the deleterious effects of acute stress and favour survival, raising the threshold at which cell death needs to be triggered.

## Data Availability

No new data were created or analyzed in this study. Data sharing is not applicable to this article.
